# Detection of submarine pipeline and cable targets based on depth feature of high resolution sonar image

**DOI:** 10.1371/journal.pone.0346343

**Published:** 2026-04-07

**Authors:** Dandan Liu, Zezhou Jin, Jiajie Chen, Zhiping Xu

**Affiliations:** 1 College of Electrical Engineering, Yancheng Institute of Technology, Yancheng, China; 2 The College of Electrical Engineering and New Energy, Hubei Provincial Engineering Technology Research Center for Microgrid, China Three Gorges University, Yichang, China; 3 School of Ocean Information Engineering, Jimei University, Xiamen, China; Northwest Normal University, CHINA

## Abstract

For Side-Scan Sonar (SSS) submarine pipeline and cable target feature extraction, there are some problems such as poor real-time performance, high false detection rate, and difficulty in deploying edge equipment.With deep feature technology,this study applies a deep neural network to detect submarine pipeline and cable targets in order to solve the above problems.To enable real-time detection of submarine pipelines and cable in SSS imagery, we improve the YOLO11n-seg model by incorporating the A2C2f and DSConv modules, leveraging the characteristic features of the target images. It reduces the false detection rate of submarine pipeline feature in SSS image,the size of parameters and realize lightweight deployment.In allusion to the Marine-PULSE submarine pipeline and cable dataset,ablation experiments and comparative experiments are designed. The experimental results show significant improvements over the original YOLO11n-seg model. Specifically,the modified model bounding box recall improved by 9.7%,and mAP@50-95 improved by 1.6%; instance segmentation recall improved by 10.3%,and mAP@50 improved by 3.6%. The detection precision and integrity are enhanced synchronously, and the size of parameters is reduced by 15%, which has stronger advantages in real-time performance. Regarding object detection, our model demonstrates superior performance, with its mAP@50 improved by 5.2% compared to YOLO12n-seg and by 12.5% compared to YOLO13n-seg. Experiments show that the model designed in this study is an effective method for real-time detection of SSS submarine pipeline and cable targets, and has a good development prospect and promotion.

## Introduction

Submarine pipeline and cable is the ‘lifeline’ of marine infrastructure,their reliability is paramount for the development of deep-sea oil and gas resources, and constitute a key pillar of the offshore petrochemical industry. [1,2]. However, in the whole process of submarine pipeline operation, geological structure changes, seawater corrosion and sediment burial will lead to pipeline leakage [3], which will cause huge economic losses and environmental pollution, and have a serious impact on society and environment [4,5]. Therefore, it is necessary to regularly carry out safety inspection and maintenance of underwater pipelines to prevent leakage accidents and reduce risks. SSS and SAS (Synthetic Aperture Sonar) [6–11] are important technologies to measure seabed topography and obtain seabed images. SAS uses a small aperture sonar transducer array to obtain higher track resolution by moving to form a virtual large aperture [12–15]. SSS is a kind of light acoustic sensor, which generally needs to be equipped with an underwater towed body for work. Its equipment is simple to install and have a high lateral resolution of the target. The target can be identified and judged by shadow [16–20]. There have been a lot of studies on autonomous detection of submarine pipelines and cables on AUV (Autonomous Underwater Vehicle) [21–24]. Compared with underwater optical images, SSS images have irreplaceable advantages in submarine pipeline and cable inspection tasks. Underwater optical imaging is severely limited by ambient light, water turbidity and suspended particles. It cannot work effectively in deep-sea lightless environments and turbid near-shore waters, which are common scenarios for submarine pipeline operation. In contrast, side-scan sonar relies on acoustic wave propagation for imaging. It is not affected by light conditions and water transparency, and can stably acquire high-resolution seabed images in full water depth and complex marine environments. In addition, side-scan sonar has a longer detection range and wider coverage. It is more suitable for long-distance and large-scale seabed pipeline inspection carried by AUVs, which fully meets the practical needs of marine engineering.

The traditional target detection and recognition method of SSS image is based on manual interpretation [25–27]. In complex terrain, it have high false detection rate and low detection efficiency [28–31]. Therefore, the online real-time detection of the target cannot be realized [32–34]. The target detection method based on deep features uses the powerful automatic feature learning ability of deep neural networks to mine effective features from massive seabed image data [35–38], improve processing efficiency and precision, which can automatically learn different scale features to achieve end-to-end automatic extraction and detection of target object features such as submarine pipelines and cables [39–41]. The use of deep structure enables the network to extract the semantics and most important features of objects in the background by gradually extracting and combining the features of different layers. It can handle more complex tasks and has higher recognition performance to meet the needs of real-time detection [42–45].

The target detection method based on deep features has two-stage method and one-stage method. The two-stage method uses a PN (Proposal Network) to search for the target object, and then uses the second network to fine-tune these suggestions and output the final prediction [46–49], mainly including R-CNN [50], Faster R-CNN [51]. Although these methods have high detection precision, they have slow detection speed and poor real-time performance [52–54], which cannot meet the needs of real-time detection of underwater AUV embedded platforms. The one-stage method mainly includes YOLO [55], SSD [56] and RetinaNet [57]. It does not need to generate candidate regions, and directly obtains the category and location of the target from the input image, with good real-time performance [58–63]. Among them, YOLO series algorithms are most widely used for their faster reasoning speed [64,65]. The core task of this study is to achieve end-to-end detection and instance segmentation of submarine pipeline targets in SSS images, and meet the real-time deployment requirements on resource-constrained edge devices of AUVs. Two-stage detection algorithms have high detection accuracy, but their inference speed is too slow to meet the needs of real-time underwater detection. Other one-stage algorithms, such as SSD and RetinaNet, have a poorer trade-off between detection accuracy and inference speed than the YOLO series. Meanwhile, these algorithms do not natively support the instance segmentation task required in this study. The YOLO series has become the most mainstream technical framework in the field of underwater sonar image target detection, and its engineering practicability has been fully verified by a large number of existing studies. We select YOLO11n-seg as the baseline model for three key reasons. First, it natively integrates the object detection branch and instance segmentation branch. It can complete both detection and segmentation tasks in a single network, which avoids additional computational overhead and deployment complexity caused by cascaded models. Second, the lightweight nano-version architecture of YOLO11n-seg has an extremely small parameter scale and computational cost, which is highly suitable for deployment on the edge computing platform of AUVs. Third, its highly modular network design provides great convenience for our targeted improvement. We can optimize the network structure for the slender linear features of submarine pipeline targets, while maintaining the lightweight and real-time performance of the model.

For the target detection algorithm for SSS submarine pipeline image, aiming at the scarcity of underwater data which is not easy to obtain [66], Du et al. [67] proposed a transfer learning framework based on GoogLeNet. Experiments show that ImageNet pre-training can improve the accuracy of the model by 10%; li et al. [68] proposed a single-stage image generation method for small sample detection to solve the problem of insufficient data. Zheng et al. [69] used CycleGAN to convert optical images into pseudo-sonar images to alleviate data scarcity. At the same time, they improved the YOLOv8 network structure, enhanced small target feature extraction through attention mechanism and deformable convolution, and improved detection accuracy significantly. Zheng et al. [70] generated synthetic data based on the principle of sonar imaging, combined with YOLOv5s to achieve zero-shot learning, and completed pipeline positioning without real labeling, with a horizontal prediction error of only 0.23 pixels.Aiming at the difficulty of underwater small target detection and the slender characteristics of submarine pipelines and cables [71], Fu et al. [72] introduced K-means++ re-clustering anchor frame to match the size distribution of small targets. At the same time, the shallow feature fusion layer is added, and the attention mechanism is combined to improve the response of small targets. The mAP@50 reaches 96.1%;cheng et al. [73] used ODConv (Omni-dimensional Dynamic Convolution) instead of traditional convolution to dynamically adapt to the target scale, and used the GAM (Global Attention Mechanism) to suppress background noise, mAP@50–95 increased by 2.51%. Wang et al. [74] designed a multi-size parallel convolution module to capture different scale features at the same time, and compared Transformer and CBAM (Convolution Block Attention Module). Finally, the AP value of 97.62% was achieved, and the inference speed was up to 100 FPS.Aiming at the problem of complex background and noise of SSS seabed image, Zhou et al. [75] proposed STGAN network, which combines Transformer to extract global features and convolution to capture local texture. A variety of loss functions are designed, and the PSNR of the image is increased by 58.73% after denoising. Lee et al. [76] combined CS (Compressed Sensing) and CoordConv network, and used coordinate information to guide denoising to achieve end-to-end training optimization nonlinear reconstruction, which can still preserve pipeline edge details in low signal-to-noise ratio scenarios. Using multi-modal fusion and cross-sensor detection can improve the detection accuracy and is suitable for complex seabed terrain. Liu et al. [77] constructed an end-to-end CNN to locate the cable directly from the magnetic anomaly data. The positioning accuracy is 30% higher than the traditional method, and the noisy data can be processed; duan et al. [78] combined SSS and SBP (Subbottom Profiler) data to enhance the weight of pipeline features through SE-Net, with a recall rate of 99.2%, which is suitable for complex seabed terrain.For AUV resource constraints, real-time airborne processing performance is more challenging [79,80]. Li et al.divided long-term sonar images into sub-images for parallel processing, and optimized the YOLOv5s network structure to achieve a detection speed of 304 ms / frame, with an AP value of 97.62%.Yang et al. [81] proposed a lightweight SS-YOLO model that enables the model to improve efficiency on edge devices with limited processing capacity and storage. In order to improve the generalization of the model and realize zero sample detection, Zheng et al. [82] generated synthetic data based on the physical model of sonar imaging, combined with YOLOv5 s to achieve zero sample detection. The horizontal positioning error in the Yellow Sea measured data is only 0.23 pixels; dakhil et al. [83] systematically compared the performance of YOLO, Faster R-CNN, U-Net and other models in sonar images, and pointed out that attention mechanism and multi-scale feature fusion are the key to improving generalization ability.In summary, the current challenges faced by the target detection task of SSS submarine pipeline image include the limited number of data set samples, high model complexity, and poor detection accuracy [84–93].

In view of the above problems, this study introduces the A2C2f (Area-Attention Enhanced Cross-Feature) based on the YOLO11n-seg network. Its adaptive weighted fusion and cross-scale optimization capabilities significantly improve the flexibility of feature expression. The DSConv (Depthwise Separable Convolution) is used to replace the standard convolution to complete the instance segmentation task of the SSS submarine pipeline, so as to ensure the precision of model detection. At the same time, the number of parameters and computing resources of the model are minimized to meet the requirements of real-time detection of underwater robots.Aiming at the target data of Marine-PULSE submarine pipeline and cable, through ablation and comparative experiments, compared with the benchmark model YOLO11n-seg, the segmentation precision mAP@50 of this research model is increased by 3.6%, the bounding boxes precision mAP@50–95 is increased by 1.6%, and the recall rate on the detection bounding boxes and instance segmentation is increased by 9.7% and 10.3%, respectively. At the same time, the number of parameters is reduced by 15%, so that the model can accurately and quickly complete the target detection task under limited computing resources, and meet the needs of AUV for underwater real-time detection.

## Baseline model

**Fig 1:**
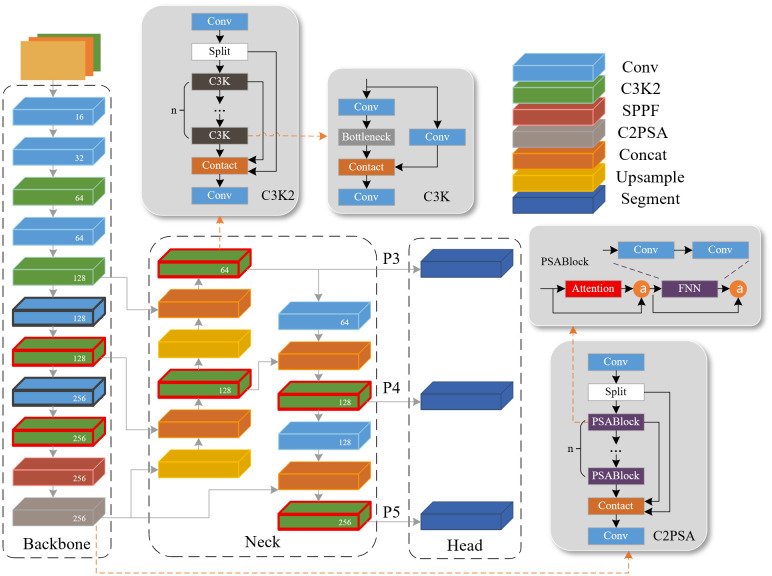
YOLOv11-seg network structure.

YOLO11 is one of the latest stable versions of the YOLO series released by Ultralytics. It is selected as the baseline framework in this study for three core reasons, which are highly matched with the requirements of underwater pipeline and cable inspection tasks. The lightweight nano version YOLO11n-seg achieves an excellent balance between detection accuracy and inference speed. Its optimized network structure has lower computational complexity and smaller parameter scale, which is fully suitable for deployment on the resource-constrained edge devices of AUVs. YOLO11n-seg natively integrates the object detection and instance segmentation branches. It can complete target positioning and contour segmentation of submarine pipelines in a single forward pass, which avoids additional computational overhead caused by multi-model cascading. Compared with other recent YOLO versions, YOLO11 shows better feature extraction efficiency for slender linear targets in low-contrast and high-noise sonar images under the same lightweight parameter scale. The SSS image of submarine pipeline target has problems such as low contrast, strong noise, and easy confusion between target and background. The YOLO11n-seg image detection algorithm can use the CNN-based target detection framework to automatically extract the depth features of the input image, such as capturing the continuous linear structure and shadow features of the pipeline, and more accurately identify the pipeline target from the complex background. The YOLOv11-seg network structure is shown in Fig 1.

It can be seen from Fig 1 that the Backbone of YOLO11-seg adopts the C3K2 module, which uses dynamic parameter adjustment to optimize shallow feature extraction and improve the calculation speed. The C3K2 module is a faster implementation of the CSP (Cross Stage Partial) bottleneck architecture with two convolutions. The CSP network divides the feature map and processes one part through the bottleneck layer, and combines the other part with the output of the bottleneck, which reduces the amount of calculation and improves the feature representation. The C3K2 module uses a smaller kernel size to make it faster while maintaining performance, so that YOLO11n-seg can extract features faster when processing images. Neck aggregates features of different resolutions and passes them to Head for prediction. The C3K2 module is used to improve the speed and performance of the feature aggregation process.

However, submarine pipelines often show a state of bending, breaking or being partially covered by sediments. In the submarine pipeline segmentation task, the fixed convolution kernel weight and rigid receptive field of the C3K2 module are difficult to adapt to the dramatic changes in the target shape, scale and background, which can easily lead to fracture and missed detection. Its conventional convolution operation has no specific sensitivity to the edge information in the image, which limits their ability to extract edge details from the image. In order to improve the detection effect of the model on the submarine pipeline target, the C3K2 module is replaced by the attention mechanism module, so that the model can accurately focus on the low-contrast target in a large number of irrelevant backgrounds, and it is required to dynamically adjust the receptive field to adapt to the irregularity of the pipeline target.

**Fig 2:**
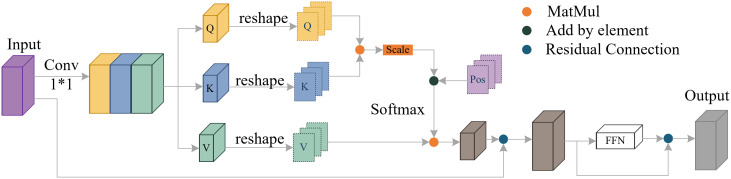
PSABlock module network structure.

YOLO11-seg enhances the feature extraction ability by adding a C2PSA module after SPPF (Spatial Pyramid Pooling – Fast), thereby improving the detection accuracy of the model. C2PSA is an extension of C2f. By introducing the PSABlock (Position-Sensitive Attention Block) mechanism, it aims to enhance the feature extraction ability through the multi-head attention mechanism and the feedforward neural network FFN (Feed-Forward Neural Network). It can selectively add residual structure to optimize gradient propagation and network training effect, whose detailed workflow is shown in Fig 2.

It can be seen from Fig 2 that the input feature map is first mapped into three independent feature matrices: query, key and value, through three parallel 1×1 convolution layers. Then, a reshape operation is performed on *Q*, *K* and *V* to split the feature maps into multiple sub-regions along the spatial dimension for efficient regional attention calculation. The similarity between *Q* and *K* is calculated via MatMul, and the result is adjusted by the attention scaling factor. The relative position bias (Pos) is added to the scaled similarity matrix, and the attention weight is obtained through Softmax normalization. The attention-weighted features are generated by matrix multiplication between the attention weight and the *V* matrix. After that, the features are processed by the FFN, and the final output of the block is obtained through two residual connections. This position-sensitive attention mechanism can effectively capture the long-distance dependence of features, enhance the model’s ability to extract the continuous linear features of submarine pipeline targets, and suppress the interference of complex seabed background noise.

Due to the limitations of high computational complexity and low energy efficiency ratio of the standard Conv module, it is not conducive to the real-time detection of underwater pipeline targets. Therefore, the lightweight Conv module should be used to replace the standard Conv module. While taking into account the detection accuracy, the number of model parameters and the amount of calculation are minimized, and the reasoning speed is improved to achieve real-time detection of underwater pipeline targets.

## Proposed method

Aiming at the problems analyzed in section 2, the YOLO11-seg network model is improved, the C3K2 module is replaced by the A2C2f module, the standard Conv module is replaced by the DSConv module, and a lightweight model for real-time detection of SSS cable targets is designed.

### A2C2f module

The A2C2 f module is constructed based on Transformer attention. The ABlock module with A2 (Area Attention) is used for residual enhancement feature extraction. The R-ELAN (Residual Efficient Layer Aggregation Networks) further enhances the optimization ability and feature expression ability of the model by introducing residual connection and new feature aggregation methods.

**Fig 3:**
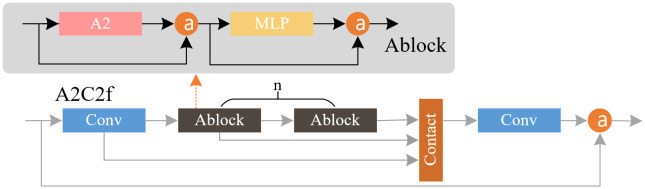
A2C2f module network structure.

Fig 3 shows the A2C2f module network structure. The core ABlock module of A2C2f includes regional attention and MLP (Multilayer Perceptron) layer, which reduces the expansion ratio of MLP in typical Transformer from 4.0 to 1.2, balances the calculation amount of attention layer and feedforward layer, and reduces the depth of stacked blocks to promote optimization, which is used for fast feature extraction and attention mechanism enhancement.

A2 uses the most direct equipartition method to divide the feature map with a resolution of (*H*, *W*) vertically or horizontally into *g* regions with a size of (*H*/*g*, *W*) or (*H*, *H*/*g*), and performs attention calculation in each region, thereby significantly reducing the computational complexity. This method realizes segmented feature processing through spatial remodeling, which not only retains the global receptive field, but also reduces the amount of calculation through simple region division operations, thus improving the speed while maintaining high performance. The large receptive field of A2 enables the model to capture the global context, combined with the position sensor to enhance the spatial information, and the activation area is more focused on the target subject.

**Fig 4:**
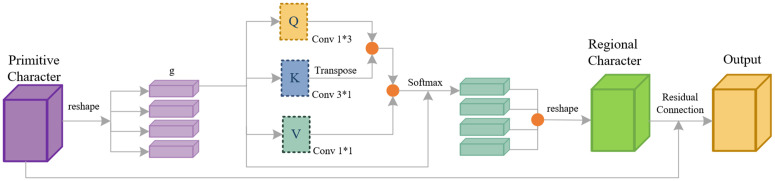
Area attention process.

The calculation process of the regional self-attention module is shown in Fig 4. The input of this module is the multi-channel feature map *X* extracted by the previous network layer, with the shape of H * W * C. Here, *H* is the height of the feature map, *W* is the width, and *C* is the number of feature channels. The query matrix *Q*, key matrix *K*, and value matrix *V* in Eq (1) are all generated from the input feature map *X* through independent linear projection layers. Each linear projection layer is implemented by a 1×1 convolution with learnable weights. The *Q* matrix is specially designed to extract the horizontal structural features of the slender linear submarine pipeline and cable targets in SSS images; the *K* matrix is used to extract the vertical structural features of the pipeline and cable targets; the *V* matrix carries the complete spatial and channel feature information of the input, and is used to generate the final attention-weighted features.

After generation, dividing *Q*, *K* and *V* into *g* equal regions along the spatial dimension respectively, following the regional division rule of the A2 attention mechanism. For the *i*-th divided region, the corresponding sub-matrices are *Q*_*i*_, *K*_*i*_ and *V*_*i*_. We define the regional self-attention calculation for the *i*-th region in Eq (1),


$$Attention(X_{i})=Softmax(\frac{Q_{i}*K_{i}^{T}}{\sqrt{d_{k}}}+B_{i})*V_{i}$$ (1)

where *d*_*k*_ is the attention scaling factor. Its value is fixed as the channel dimension of the single-head attention feature, which is determined by the channel number of the generated *Q* and *K* matrices. It is used to avoid the gradient disappearance of the Softmax function caused by excessive inner product values of *Q* and *K*. *B*_*i*_ is the relative position bias matrix within the *i*-th region. It is a learnable parameter initialized before model training, and is updated synchronously during the end-to-end training process. It encodes the spatial position information of pixels in the region, to help the model capture the continuous linear structure of submarine pipeline targets. Softmax is the standard normalization function. It normalizes the similarity score between *Q*_*i*_ and *K*_*i*_ into attention weights ranging from 0 to 1, to realize adaptive weighting of target-related features and background features.

After completing the attention calculation for all regions, concatenating the attention output of each region along the spatial dimension, and perform residual connection with the original input feature map X. The final output of the regional attention module is defined in Eq (2),


$$Output=X+Concat(Attention(X_{1}),...,Attention(X_{g}))$$ (2)

In Eq (2), *X* represents the original input feature map of the A2 regional attention module, and Concat represents the feature concatenation operation. The residual connection structure helps to optimize gradient propagation during model training.The computational complexity of each region is $(H*W/g{)}^{2}*C$, and the total complexity is $g*(H*W/g{)}^{2}*C$, which is *g* times lower than the global self-attention *H*^2^ * *W*^2^ * *C*, and enhances the spatial expression ability of features at low computational cost.

The core idea of R-ELAN is to improve the gradient flow and feature expression ability of the model through the improvement of residual connection and feature aggregation method. The application layer scaling for each region does not solve the optimization challenge, but increases the delay. At the same time, the aggregation method is redesigned. The original ELAN layer first processes the input through the transition layer, and then divides it into two parts for processing and splicing. R-ELAN first generates a single feature map by adjusting the channel dimension through the transition layer, and then splices after subsequent module processing to form a bottleneck structure.

### DSConv module

**Fig 5:**
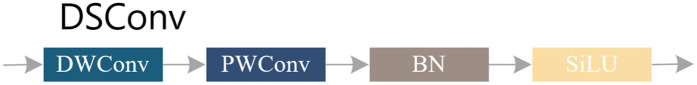
DSConv module network structure.

Fig 5 shows the DSConv module network structure.DSConv consists of DWConv (Depthwise Convolution) and PWConv (Pointwise Convolution). DWConv is used to extract spatial features and PWConv is used to extract channel features.

**Fig 6:**
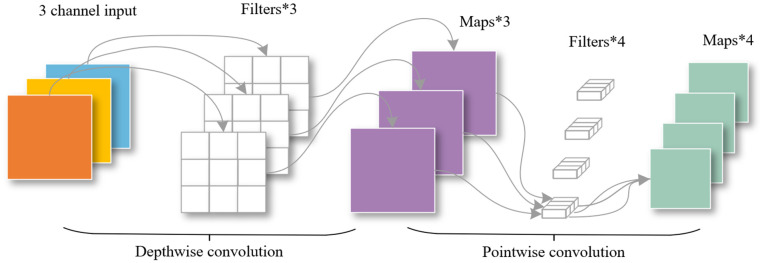
DSConv module process.

DSConv first performs DWConv on each channel, and then merges all channels through PWConv as the output feature map, so as to reduce the calculation amount and improve the calculation efficiency. After that, adding activation function and BatchNorm helps to improve the nonlinear expression ability of the network, so that the network has a stronger ability to fit more complex functions. The DSConv module process is shown in Fig 6.

The operational workflow of DSConv is illustrated in the Fig 6. Taking the 3-channel input feature map in the example as reference, DWConv first applies an independent convolution kernel to each single input channel, and extracts spatial features separately for each channel without cross-channel feature interaction. Then, PWConv adopts 1×1 convolution kernels to process the output feature maps from DWConv, which completes the fusion of cross-channel features and adjusts the number of output channels. Compared with standard convolution, DSConv significantly reduces computational complexity and parameter count, which is more suitable for lightweight model deployment on resource-constrained edge devices.

Assuming that the size of the input feature map is $D_{k}*D_{k}*M$ and the size of the convolution kernel is $D_{f}*D_{f}*M$, the total amount of calculation for *N* convolutions is $D_{k}*D_{k}*D_{f}*D_{f}*M*N$; the total amount of DWConv calculation is $D_{k}*D_{k}*D_{f}*D_{f}*M$; the total amount of PWConv is $M*N*D_{k}*D_{k}$; the total amount of calculation of DSConv is $D_{k}*D_{k}*D_{f}*D_{f}*M+M*N*D_{k}*D_{k}$. Calculating the ratio of the computational load of DSConv to standard convolution, and the result is shown in Eq (3),


$$\frac{D_{k}*D_{k}*D_{f}*D_{f}*M+M*N*D_{k}*D_{k}}{D_{k}*D_{k}*D_{f}*D_{f}*M*N}=\frac{1}{N}+\frac{1}{D_{k}^{2}}$$ (3)

In general, *N* is large, and $\frac{1}{N}$ can be ignored. *D*_*k*_ represents the size of the convolution kernel. The parameters and calculations of DSConv are reduced to the original $D_{k}^{2}$, so as to obtain faster speed, easier to transplant, and can achieve high-precision operations on smaller devices.

### Lightweight network model

**Fig 7:**
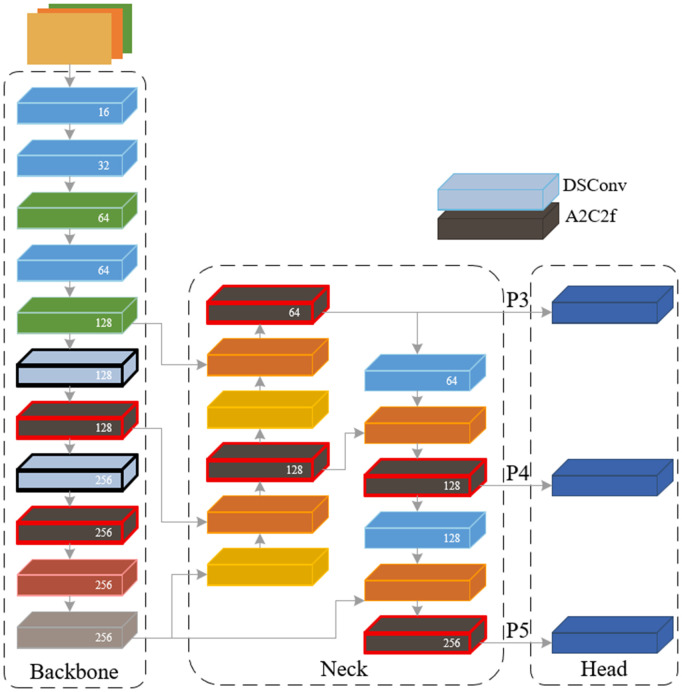
The improved lightweight network model structure of YOLO11-seg network model.

It can be seen that the A2C2f module based on the regional attention mechanism can focus more on the target body for the characteristics of slender, curved and low resolution of the submarine pipeline target. Its deformable convolution can adjust the sampling point along the pipeline direction, which can improve the low detection accuracy of the submarine pipeline target and the problem of missed detection. Therefore, the original C3K2 module in the YOLO11-seg network model is replaced by the A2C2f module. Fig 7 shows the improved lightweight network model structure of YOLO v11-seg network model.

It can be seen from Fig 7 that the C3K2 module focuses on feature parallelism. By replacing the C3K2 module with the A2C2f module, its multi-convolution kernel is used to capture multi-scale, and the A2 is embedded in the middle layer to focus on the key area earlier. Aiming at the characteristics of SSS submarine pipeline target slender and blurred edges, the detection accuracy is improved and the missed detection rate is reduced. In the occlusion and low contrast scene, the target feature is extracted by regional attention priority to improve the integrity of segmentation. At the same time, the DSConv module is introduced, designed to achieve a more lightweight architecture by reducing both the parameter count and computational complexity. It greatly reduces the computational cost while retaining the global receptive field.

The P3 layer is a shallow network, and the receptive field is relatively small. It generally contains more location and detail information. At the end of this stage, the A2C2f module is added to process the multi-scale feature weight distribution relationship and enhance the transmission of edge and texture features. At the same time, by weighting each channel, the attention to the useful detail feature channel is increased, which is helpful for the recognition of the submarine pipeline target by the small target detection head after the P3 layer feature fusion.

The P4 layer is used as the middle layer network. At the end of this stage, the A2C2f module is introduced. At the same time, the seabed image is divided into four regions, and the attention calculation is performed in each region. The receptive field of the large target covers multiple regions. The localization of the regional attention has little effect on its global semantic information. It can pay more attention to the spatial location of the submarine pipeline target, suppress the background features of the unimportant seabed image, reduce the propagation of invalid features, improve the positioning accuracy, and reduce the calculation amount by 75% while completing the high-quality feature aggregation and avoiding the problem of feature redundancy.

### Real-time detection method process

**Fig 8:**
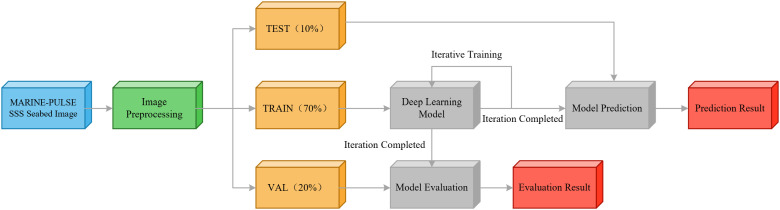
The network model detection method flow, the network iterative training, get the model prediction and evaluation results.

The end-to-end workflow of the proposed lightweight model for real-time SSS pipeline and cable target detection is shown in Fig 8. The whole workflow includes four core steps: image preprocessing, dataset splitting, model iterative training, model prediction and performance evaluation.

Image preprocessing is the foundational step for model training and inference. This step is performed on all SSS images before network input. The preprocessing pipeline is designed specifically for the characteristics of SSS submarine pipeline images, with detailed operations as follows,

All input SSS images are uniformly resized to a fixed resolution of 640 × 640 pixels. This resolution matches the input dimension requirement of the improved YOLO11n-seg model. The original aspect ratio of each image is maintained during resizing. Zero-padding is applied to fill blank areas, which avoids shape deformation of the slender linear pipeline and cable targets. SSS images inherently contain strong speckle noise caused by acoustic wave scattering in seawater. Gaussian filtering is adopted for image smoothing. This operation effectively suppresses background noise, while retaining the edge details and linear structural features of pipeline and cable targets.

After the completion of image preprocessing, the preprocessed original images and the augmented images generated by data enhancement are integrated. The complete dataset is then randomly divided into training set (70%), validation set (20%) and test set (10%) according to a fixed ratio of 7:2:1. No data overlap exists between the three sets, and the distribution of target features remains consistent across different sets. The training set is used for model weight learning. The validation set is applied to monitor overfitting risk during the training process. The test set is adopted for the final unbiased performance evaluation of the model.

## Experimental evaluation

### Dataset

The Marine-PULSE dataset is the first public SSS dataset for marine engineering geology in the Bohai Sea in China. It was created by Du Xing [66] and his colleagues in the field of marine geological disasters. It contains various common SSS seabed image data that appear in the Yellow River estuary. It is used to automatically identify marine engineering structures and common seabed landforms. It covers four typical targets: submarine pipelines (323), seabed residual deposits (134), seabed surface (88), and engineering platform legs (82). The data source includes a variety of advanced sonar equipment such as EdgeTech4200FS, BenthosSIS-1624, etc., thus ensuring the diversity and reliability of the data. The subject selects the SSS submarine pipeline image of the submarine pipeline (323), a total of 323 pictures, and the size of each picture is 640 × 640 pixels.

The actual scene of submarine pipelines and cables is complex and changeable. The factors such as the shape and position of pipelines and cables, lighting conditions, water quality and so on will affect the image presentation. It is difficult to include all possible situations only by the original data set. Data enhancement will perform operations such as rotation, flipping, scaling, and adding noise to the original image, creating more images with different perspectives, lighting conditions, and background interference to improve data diversity. The multi-type data augmentation strategy adopted in this study effectively alleviates the overfitting problem caused by limited original labeled samples, and improves the generalization performance of the model.The 323 underwater acoustic images with annotations in the data set are enhanced to 1615. Let the model learn more features and improve the generalization ability and robustness of the convolutional neural network.

In the training submarine pipeline target detection task, the data set is divided into Train, Val and Test according to the ratio of 7: 2: 1, which can reasonably evaluate the performance of the model, understand the status of each period of the model, quickly find out the problem and correct it.

### Ablation experiment

In this study, the A2C2f module was used to replace the C3K2 module, and the DSConv module was used to replace the Conv module. To directly evaluate the impact of the proposed modules on the SSS pipeline detection model, we conducted ablation experiments. In the original YOLO11n-seg, the C3K2 module is replaced by the A2C2f module, and the Conv module is replaced by the DSConv module. After the data is enhanced, the Marine-PULSE dataset is trained and compared. The ablation experiment result can be given in Table 1.

**Table 1:** Ablation experiment result.

Model	BOX/%			MASK/%			Model Scale	
	R	mAP@50	mAP@50-90	R	mAP@50	mAP@50-90	Parameters	GFLOPs
YOLO11n-seg	67.1	81.3	56.2	60.7	66.8	30.0	2.8	10.2
YOLO11n-seg + A2C2f	67.0	82.6	57.0	59.8	68.1	30.3	2.8	10.2
YOLO11n-seg + DSConv	70.5	78.1	53.9	62.4	66.8	29.8	2.3	9.4
The Research Model	76.8	81.8	57.8	71.0	70.4	30.3	2.4	9.6

From Table 1, it can be seen that the first group was the baseline model YOLO11n-seg; in the second group, the A2C2f module is introduced, and the accuracy of the detection frame is improved by 3.0%, which effectively reduces the false detection. Both mAP@50 and mAP@50-95 are improved, which verifies the optimization of high IoU detection accuracy. In terms of segmentation performance, both mAP@50 and mAP@50-95 are improved, the segmentation fineness is significantly enhanced, the capture of edges and details is better, and the number of parameters is slightly increased. A2C2f optimizes the feature expression at a minimal cost, which is more suitable for scenes with high detection accuracy requirements. The third group introduced the DSConv module, the recall rate increased by 3.4%, significantly improved, reflecting the ability of less missed detection, the accuracy rate decreased, indicating that the false detection increased, in the segmentation performance, the recall rate increased, mAP@50-95 decreased slightly, indicating that more real targets can be detected, but the high fineness was slightly sacrificed, the number of parameters decreased significantly, reflecting the DSCconv is efficient convolution, greatly reducing the computational cost. The fourth group introduces the A2C2f module and the DSConv module, and adopts the model for this study. On the bounding box, the recall rate is increased by 9.7%, and the missed detection rate is significantly reduced, which is the most obvious improvement among the three groups. Both mAP@50 and mAP@50-95 are improved to maintain high positioning accuracy. In terms of segmentation performance, the recall rate and detection accuracy are greatly improved, and the segmentation accuracy and integrity are improved simultaneously. The amount of parameters and calculations are reduced to achieve lightweight.

A2C2f focuses on accuracy, and DSConv focuses on efficiency. After the fusion of the two, the optimal trade-off is achieved in the scenario of missed detection sensitive and real-time operation. This study uses the model to achieve the improvement of detection accuracy and the leap of recall rate at a lower computational cost. For the SSS submarine pipeline target, a better balance between model complexity and detection accuracy is achieved, which can meet the real-time detection requirements of SSS equipment.

Gradient ablation experiments are conducted to verify the dependence of the proposed model’s performance on the size of training data. Under the premise of fixed validation set and test set, the original labeled images in the training set are randomly sampled at ratios of 10%, 30%, 50%, 70% and 100% for model training. All experimental groups adopt the same data augmentation strategy and training hyperparameters to ensure the uniqueness of control variables. The results show that the model’s detection and segmentation performance improves steadily with the increase of training samples, and it maintains stable and excellent performance under small training sample conditions. When the training sample size reaches 50% of the original training set, the model achieves more than 95% of the performance under the full training set, which is close to the optimal performance level. Even when only 30% of the original training samples are used, the model still retains more than 90% of the full training set performance for both bounding box detection and instance segmentation tasks. These results confirm that the proposed method has low dependence on the size of the labeled dataset. It has strong adaptability and engineering application value for practical underwater inspection scenarios where high-quality labeled sonar images are difficult to obtain in large quantities.

### Loss function

The total loss of the proposed model consists of two core parts: the detection branch loss and the instance segmentation branch loss. The final total loss is calculated as the weighted sum of each sub-loss term, as shown in Eq (4),


$$L_{total}=\lambda_{1}L_{det}+\lambda_{2}L_{mask}$$ (4)

In Eq (4), *L*_*det*_ represents the detection branch loss, *L*_*mask*_ represents the instance segmentation mask loss. $\lambda_{1}$ and $\lambda_{2}$ are the weight coefficients of each loss term, which are set to 1.0 and 0.5 respectively in this study. The weight settings are consistent with the default hyperparameters of the YOLO11 framework, to achieve balanced optimization of detection and segmentation tasks.

The detection branch loss *L*_*det*_ is composed of three sub-loss terms, and its calculation formula is shown in Eq (5),


$$L_{det}=L_{cls}+L_{box}+L_{obj}$$ (5)

The detection branch loss *L*_*det*_ consists of three sub-terms: the classification loss *L*_*cls*_, bounding box regression loss *L*_*box*_, and objectness loss *L*_*obj*_. Among them, *L*_*cls*_ and *L*_*obj*_ are both calculated by Binary Cross-Entropy (BCE) loss, where *L*_*cls*_ measures the error between the predicted category probability and the ground truth label to optimize the classification accuracy of pipeline and cable targets, while *L*_*obj*_ evaluates the confidence of target existence in the predicted anchor box to reduce the false detection rate in complex seabed backgrounds; *L*_*box*_ is computed with Complete Intersection over Union (CIoU) loss, which comprehensively considers the overlap area, center point distance and aspect ratio between the predicted bounding box and the ground truth box, to improve the positioning accuracy of the target bounding box.

The instance segmentation branch loss *L*_*mask*_ uses pixel-level Binary Cross-Entropy loss. It calculates the classification error of each pixel between the predicted mask and the ground truth mask. This loss term optimizes the contour segmentation integrity of pipeline and cable targets, especially for bending, fractured and partially sediment-covered targets.

### Contrast experiment

To comprehensively evaluate the performance of our proposed model, we conducted comparative experiments with other networks, and maintained the comparison under the Marine-PULSE data set and 260 training rounds after data enhancement. The contrast experiment result can be given in Table 2.

**Table 2:** Contrast experiment result.

Model	BOX/%		MASK/%		Model Scale	
	mAP@50	mAP@50-90	mAP@50	mAP@50-90	Parameters	GFLOPs
YOLO11n-seg	81.3	56.2	66.8	30.0	2.8	10.2
YOLO12n-seg	76.6	52.4	57.9	25.4	2.8	10.2
YOLO13n-seg	80.9	57.8	68.0	30.4	2.7	10.1
The Research Model	81.8	57.8	70.4	30.3	2.4	9.6

**Fig 9:**
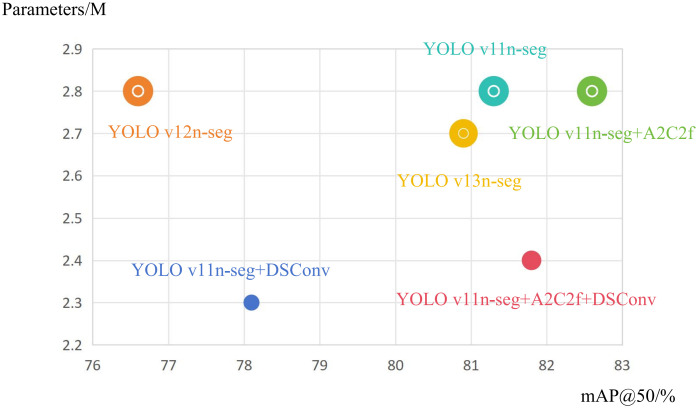
The radius of the circle represents GFLOPs by comparing the mAP@50 and Parameters of different models.

From Table 2, it can be seen that the YOLO11n-seg + A2C2f + DSConv model proposed in this study has improved mAP@50 compared with other models in terms of detection frame and segmentation performance.mAP@50-95 is the same as YOLO13n-seg, surpassing YOLO11n-seg and YOLO12n-seg. At the same time, the number of parameters is reduced by 15%, and GFLOP is reduced by 6%. Fig 9 is the comparison between the average accuracy of different models and the number of parameters. The lightweight range is far more than other models, and the deployment cost is lower, which is more suitable for edge devices and real-time scenarios.

The processing time of each method can be given in Table 3.

**Table 3:** Comparison of the average Preprocessing, Inference, and Postprocess time per image for model evaluation of the validation set for different models.

	YOLO11n-seg	YOLO12n-seg	YOLO13n-seg	The Research Model
Preprocessing/ms	3.9	3.6	3.5	2.8
Inference/ms	9.1	10.2	10.1	9.1
Postprocess/ms	4.4	4.4	3.0	2.4
FPS	57	59	60	70

From Table 3, it can be seen that the model proposed in this study has a significant improvement in the number of frames per second FPS (Frames Per Second). With its superior real-time processing capability, the model is particularly suitable for applications that require high-speed operation, thus effectively supporting real-time detection on edge devices.

### Overfitting analysis and robustness evaluation

Overfitting is a common challenge for deep learning models, especially in tasks with limited labeled samples. This study adopts multiple targeted strategies to mitigate overfitting and fully verifies the model’s generalization ability through quantitative experiments: a strict non-overlapping dataset division and real-time training monitoring mechanism are established, and the synchronous stable convergence of training and validation loss curves confirms no serious overfitting occurs during training; multi-type data augmentation expands the original 323 labeled images to 1615 effective training samples, enriching data diversity to improve generalization performance; the lightweight design reduces model parameters by 15% compared with the baseline YOLO11n-seg, lowering fitting complexity and overfitting risk. Gradient dataset ablation experiments further prove that the model maintains over 95% of full-dataset performance with only 30% of original labeled samples, showing excellent anti-overfitting ability and generalization in small-sample scenarios.

Robustness is a critical index for the practical engineering application of models in complex underwater environments. The proposed method exhibits strong robustness in multiple dimensions, which is fully verified by module design and quantitative experiments: the A2C2f module with regional attention mechanism effectively suppresses seabed background noise interference, achieving 9.7% and 10.3% improvements in bounding box and instance segmentation recall respectively compared with the baseline model; the model maintains excellent detection and segmentation performance for pipeline targets with bending, fracture or partial sediment coverage; it also shows stable performance under different training set scales, with smaller performance degradation than baseline models when training samples are reduced. Meanwhile, the model achieves an end-to-end processing speed of 70 FPS, maintaining stable real-time performance on resource-constrained AUV edge devices to meet the requirements of actual underwater inspection tasks.

## Conclusion

In this study, aiming at the difficulties of SSS submarine pipeline target detection, based on the depth feature extraction technology, the YOLO11n-seg is improved by using A2C2f module and DSConv module to realize the autonomous detection of SSS submarine pipeline target.Compared to YOLO11n-seg, our model shows consistent improvements: segmentation mAP@50 and bounding box mAP@50-95 improved by 3.6% and 1.6%, respectively. Meanwhile, the recall rates for detection bounding boxes and instance segmentation improved by 9.7% and 10.3%, respectively. At the same time, the number of parameters is reduced by 15%, and the deployment cost is lower, which is more suitable for edge devices and real-time scenarios. It has significant engineering application value in real-time SSS submarine pipeline and cable target processing. It provides a feasible and reliable technical solution for marine survey, pipeline and cable target feature extraction and other tasks, and has a good development prospect and promotion.

There are still many aspects to be improved in this research model. For example, using style transfer to increase high-level features, further enriching data content, how to improve the module in real-time detection scenarios, so that it can further improve the detection accuracy and accuracy while maintaining lightweight will be the next research direction.
